# Dietetics: Its Bearing on Special and General Tissue Building

**Published:** 1888-07

**Authors:** W. J. Thayer

**Affiliations:** Brooklyn, N. Y.


### DIETETICS: ITS BEARING ON SPECIAL AND GENERAL TISSUE BUILDING.

W. J. Thayer, D. D. S., M. D., Brooklyn, N. Y.

For many years there was no subject that gave the writer so much solicitude as how best to treat the asthenic patient dietetically. There can be no question of the great utility of dietetic treatment in various diseases. For instance, in diabetes, consumption, typhoid, and typhus fevers, and other grave pathological conditions. Reconstructives for convalescence. Nutritive supports for the adynamic. Beef tea has been found of but little value as a supporter. As a builder, almost worthless.

The soft tissues of the body, and even the bones, can be repaired, built over, and new scar tissue formed, but not so with certain other tissues, the enamel, dentine, and cementum. Therefore, when these three petrous tissues are formed, *they are built up forever!* It follows, then, that the way in which, or *result* of building, is an exceedingly important matter.

Are the petrous tissues of any value, at least as comminuters of food, articulators of speech, and for contour of features? If they are, then it is the bounden duty of every one to so assist them in their construction that they will be able to resist the greatest amount of disintegrating influences. But they who are to be most benefited do not know their need at the proper time. Therefore it devolves upon others to assist them to build properly, and upon no class of individuals is this duty so strongly imposed as upon the physician.

There is a vast difference in the construction and density of the teeth in different individuals.

There is also a vast difference in the ability of the teeth of different individuals to *resist decay!*

For these two conditions there is a cause. There are but a few children that have arrived at the age of ten years that can show a perfectly sound set of six-year molars. Why? The six-year molars—the first molars of the *permanent* set—are the largest of the back teeth, and of *immense value!* Many people think it's of no consequence to lose a back tooth. One had better lose two incisors, rather than a molar, or bicuspid, even.

The teeth are composed of *soft solids* and *calcareous salts.* The latter constituents give the teeth their ability to *resist* decay! Human teeth, for the past three decades, have been increasing in softness and effeminacy to an alarming extent. Why? Simply because they have been starved out of existence! *They have been deprived of their lime salts!* Starved! He who eats can hardly fail to get a liberal amount of nitrogeneous matter, and that pabulum will supply the soft protoplast of tooth structure, as well as of all soft tissues. But the proteins —albuminoids or nitrogenous matter—will *not* supply to the teeth their inorganic constituents any more than a diet of cellulose and sawdust.

All tissue *building,* all tissue *support,* must come from *appropriated* pabulum. Suppose that the nutriment matter *to be appropriated* contains but little or none of the calcareous salts, where, pray, are the petrous tissues, the bones, and certain constituents of muscular tissue to get their supply?

The cereals contain this supply of inorganic constituents if, and provided, they are not "civilized" beyond Providential intention, by being *bolted into nothingness! "Bolted!"*

It is the outside of all our grains—less their coarser woody fibres—that are rich in carbonate and phosphate of lime. Tooth builders! Bone constructors! Muscle densi-*fiers!* A glorious triad! The only food that contains a *sufficient* supply of these lime salts that has been arranged by a Superior Wisdom for easy human digestion and appropriation.

Let us repeat, "*That teeth once built up, are built up forever!*" The time to pack the lime salts is *when they are building!*

The teeth begin to form as early as the sixth week of fœtal existence. The expectant mother ought to partake of the *coarse* bread foods *three times each day. Oatmeal, Graham bread, rye and Indian bread, corn bread, rye bread*—all constructed out of the *unbolted* product of grain used.

If the child is brought up on human milk, then the mother or nurse should eat liberally of these same foods. It is through the umbilical cord and mammary gland that the greatest amount of good can be done for the petrous tissues.

The permanent teeth commence to form before birth and so continue till the wisdom teeth are erupted. Therefore, the babe, child, youth, and he of mature years ought to be advised and directed to *pack* away, for future use, these petrous tissue builders.

"How shall they hear without a preacher?" And who can expatiate upon the text like him who has been commissioned by High Heaven to heal the sick—the physician?

Flabby muscular tissue and atony of the womb will be found due to a want of a normal supply of the lime salts in their texture. In well-developed muscular tissue there is from twenty to twenty-five per cent. of inorganic constituents. Thus it appears that not only the petrous tissues *demand* these lime salts, but many other textures of the human body are vastly benefited by such constituents, furnished to them in a physiological manner and *not* fed pharmaceutically.

Our grandfathers, who were brought up on the coarse grain foods, did not lose their teeth before a very late period in life, yet they had as many microbes to contend with as do they who to-day content themselves on gluten and raw starch in *all* of their bread foods. They, in the absence of bolting mills, *did partake of the whole of the grain*, and the results are patent to-day in their teeth!

Many children, by force of circumstances, are brought up on the bottle. Cow's milk is substituted for the maternal breast. But the tough casein found in cow's milk cannot be as easily disposed of as the easier digestive casein of human milk. There are children by the score that cannot digest it at all. In such cases it ought to be partly pre-digested with pancreatin, but few mothers or nurses can attend to this properly. The digestion will be carried too far and the milk made bitter; or too much heat applied and the digestive ferment ruined altogether, so that there will be no digestion of the milk whatever, and the digestive apparatus of the child taxed more severely.

If this is true of good country milk, what shall we say for those children that are compelled to subsist upon such milk as is dealt out to them in large cities?

A good, *evenly balanced*, *reliable*, and *easy digesting* artificial food for infants is much superior to indifferent maternal nursing, or doubtfully contesting the tough casein found in cow's milk. That there are artificial foods possessing those qualities, the subjoined table, transcribed from the *Pharmacuet*, Central Halle, 1886, Berlin, will show. Particular attention is directed to the proteins, or nitrogenous matter, the salts and inorganic substances, to the lime and phosphoric acid in those substances, and to the *ready digestibility* of the different foods.

In Mellin's food the digestibility is represented to be as low as 7.38 per cent., while Carnrick's soluble food is the *easiest* of *digestion*, it being 16.45 per cent.

Carnrick's food has the highest per cent. of fat, and 18.22 per cent. of proteins, or nitrogenous matter. Anglo-Swiss, the next highest in proteins, 12.38. The amount of nitrogenous matter and ease of digestion are two important matters in an artificial food for infants.

It is for the petrous tissues that we plead. The highest amount of "salts and inorganic constituents" is represented as 3.53 per cent., but the inorganic constituents, containing *lime* and *phosphoric acid* (see table), are unbalanced, they being of lime 0.155, and phos. acid 0.583.

Carnrick's soluble food has of the "inorganic constituents" 2.991 per cent., and of *lime*, 0.645; of *phosphoric acid*, 0.874 per cent., which shows this food to be the best as a petrous tissue builder. Anglo-Swiss comes next. See table.

The starch in any of these foods should be converted into *dextrine* before they are compounded together, or ingested. The amyolytic ferments of the saliva, pancreatic, and intestinal juices will easily convert dextrine into maltose, which is ready for immediate absorption. If, however, the artificial food is carried beyond dextrine in the process of manufacture, into a malt sugar, maltose, then the secretions of the stomach are liable to cause a ferment of the malt sugar *before* it can reach the duodenum, and a *sour* or *vinous fermentation* continues through the digestive tract and defeats the objects sought.

The writer agrees with Dr. Biegler (see HOMŒOPATHIC PHYSICIAN, March, 1888, page 107), "People are apt to begin

| AN ANALYSIS OF EIGHT OF THE MOST COMMON ARTIFICIAL FOODS FOR INFANTS. | Carnrick's Soluble Food. A Milk Food. | Nestle's Food. A Milk Food. | Anglo-Swiss Milk Food. A Milk Food. | Horlick's Food. A Malt Food. | Mellin's Food. A Malt Food. | Wells, Richardson & Co.'s Food. A Starch Food. | Dr. Ridge's Food. A Starch Food. | Imperial Granum. A Starch Food. |
|---|---|---|---|---|---|---|---|---|
| Fat, | 5.00 | 4.66 | 2.37 | 0.60 | 0.50 | 2.19 | 1.27 | 0.80 |
| Protein substances, Albuminoids, Nitrogenous, | 18.22 | 11.46 | 12.38 | 11.30 | 8.34 | 9.05 | 8.76 | 10.73 |
| Hydro-Carbons, Dextrine, etc., | 67.74 | 76.69 | 76.03 | 79.04 | 79.29 | 78.44 | 80.45 | 78.88 |
| Cellulose, | . . . | 0.10 | 1.09 | 0.55 | 0.58 | 1.54 | 0.73 | 0.97 |
| Water, | 6.14 | 5.34 | 6.18 | 5.75 | 7.76 | 6.52 | 8.31 | 8.25 |
| Salts and inorganic constituents, | 2.991 | 1.75 | 1.95 | 2.76 | 3.53 | 2.26 | 0.48 | 0.37 |
| Amount of Nitrogen in protein substances, | 2.915 | 1.833 | 1.981 | 1.809 | 1.335 | 1.448 | 1.403 | 1.717 |
| Amount of protein substances READILY DIGESTIBLE, | 16.45 | 11.09 | 11.20 | 10.85 | 7.38 | 8.35 | 7.97 | 9.55 |
| Proportion of nitrogenous alimentary substances. Proteins=1, | 1:4.4 | 1:7.7 | 1:6.6 | 1:7.1 | 1:9.6 | 1.92 | 1:9.3 | 1:7.5 |
| The inorganic constituents contain of { Lime, | 0.645 | 0.390 | 0.520 | 0.076 | 0.155 | 0.390 | 0.060 | 0.001 |
| The inorganic constituents contain of { Phosphoric Acid, | 0.874 | 0.630 | 0.800 | 0.421 | 0.583 | 0.688 | 0.260 | 0.167 |

*too early* to feed their children with starchy food, and to withhold milk."

Starches, as such, will not digest in an infant's digestive apparatus, only to a very limited extent, for the reason that the amyolytic ferment found in the saliva, pancreatic, and intestinal juices is not secreted in sufficient quantities to convert the raw starch into soluble sugar, and the result can only be that these hydro-carbons rasp and scrape down the alimentary track, denuding it of its epithelium, establishing blenorrhœas and serious bowel lesions.

Starches that are fed to infants should be baked at a temperature of 350° F. for *not less* than eight hours. This will convert the starch into *dextrine*, which *will not ferment* in the stomach, and when it reaches the duodenum the amyolytic ferments immediately convert it into maltose, or soluble sugar, and it is ready for immediate absorption and has had *no time* or *condition* to ferment!

The tough casein of cow's milk should also be partly pre-digested, so as to avoid insoluble, cheesy curds. All this is performed in a first-class artificial food for infants, as is *plainly indicated* by the above table.

As soon as one is aware that his services will be required for an accouchement, he will find that by requiring his patient to eat liberally of the above-mentioned bread foods that he is infiltrating the parenchyma of uterine tissue with a substance that will do much to prevent atony of that organ, and promote a physiological full term and useful normal contractions.

